# An artificial neural network model based on autophagy-related genes in childhood systemic lupus erythematosus

**DOI:** 10.1186/s41065-022-00248-7

**Published:** 2022-09-16

**Authors:** Jinting Wu, Wenxian Yang, Huihui Li

**Affiliations:** 1 Department of Oncology, The First Affiliated Hospital of Wenzhou Medical University, Wenzhou, Zhejiang People’s Republic of China; 2 Pediatrics, Shaoxing University School of Medicine, Shaoxing, 312000 China; 3 Department of Breast Surgery, The First Affiliated Hospital of Wenzhou Medical University, Wenzhou, 325006 Zhejiang People’s Republic of China

**Keywords:** Autophagy, Artificial neural network, cSLE, Immune cell infiltration

## Abstract

**Background:**

Childhood systemic lupus erythematosus (cSLE) is a multisystemic, life-threatening autoimmune disease. Compared to adults, SLE in childhood is more active, can cause multisystem involvement including renal, neurological and hematological, and can cause cumulative damage across systems more rapidly. Autophagy, one of the core functions of cells, is involved in almost every process of the immune response and has been shown to be associated with many autoimmune diseases, being a key factor in the interplay between innate and adaptive immunity. Autophagy influences the onset, progression and severity of SLE. This paper identifies new biomarkers for the diagnosis and treatment of childhood SLE based on an artificial neural network of autophagy-related genes.

**Methods:**

We downloaded dataset GSE100163 from the Gene Expression Omnibus database and used Protein–protein Interaction Network (PPI) and Least Absolute Shrinkage and Selection Operator (LASSO) to screen the signature genes of autophagy-related genes in cSLE. A new artificial neural network model for cSLE diagnosis was constructed using the signature genes. The predictive efficiency of the model was also validated using the dataset GSE65391. Finally, "CIBERSORT" was used to calculate the infiltration of immune cells in cSLE and to analyze the relationship between the signature genes and the infiltration of immune cells.

**Results:**

We identified 37 autophagy-related genes that differed in cSLE and normal samples, and finally obtained the seven most relevant signature genes for cSLE (DDIT3, GNB2L1, CTSD, HSPA8, ULK1, DNAJB1, CANX) by PPI and LASOO regression screening, and constructed an artificial neural network diagnostic model for cSLE. Using this model, we plotted the ROC curves for the training and validation group diagnoses with the area under the curve of 0.976 and 0.783, respectively. Finally, we performed immunoassays on cSLE samples, and the results showed that Plasma cells, Macrophages M0, Dendritic cells activated and Neutrophils were significantly infiltrated in cSLE.

**Conclusion:**

We constructed an artificial neural network diagnostic model of seven autophagy-related genes that can be used for the diagnosis of cSLE. Meanwhile, the characteristic genes affect the immune infiltration of cSLE, which may provide new perspectives for the exploration of cSLE treatment and related mechanisms.

**Supplementary Information:**

The online version contains supplementary material available at 10.1186/s41065-022-00248-7.

## Introduction

Systemic lupus erythematosus (SLE) is a highly heritable, relapsing autoimmune disease with multiple clinical and laboratory manifestations [1–3].Compared to adults, SLE in children has a worse outcome and can be complicated by multiple systemic lesions (neuropsychiatric lupus, lupus nephritis, macrophage activation syndrome, etc.) early in the disease [4, 5]. The pathogenesis of SLE remains elusive, involving multiple aspects such as genetic risk factors, epigenetic mechanisms, and environmental infection triggers. As we know previously, some environmental factors such as UV can exacerbate or induce SLE, while others can be involved in SLE by altering epigenetics such as altered DNA methylation, and histone phosphorylation [6]. In addition, variants in some genes are closely associated with SLE, such as those carrying *C1QA* purely pathogenic alleles and those carrying *PEPD* purely pathogenic alleles [7]. Currently, genome-wide association studies have identified at least 70 lupus susceptibility loci [8]. It is worth noting that even in cSLE without any permanent functional impairment, the 10-year survival rate is only 66.1%, which is enough to draw our attention to SLE in children [9].

Autophagy, one of the core functions of cells, includes three main modalities: microautophagy, macroautophagy and chaperone-mediated autophagy (CMA). Current studies have revealed the involvement of autophagy in the progression of various autoimmune diseases of the body, such as systemic lupus erythematosus, rheumatoid arthritis, desiccation syndrome and antiphospholipid antibody syndrome [10]. With the rapid development of genome-wide association studies and animal models, the mechanism of the role of autophagy in SLE has been gradually elucidated. TIM-1 was found to regulate autophagy and thus alleviate the damage of lupus nephritis podocytes [11]. Animal experiments have shown that WDFY4 can effectively improve SLE by regulating the behavior of autophagic B cells [12]. Autophagy-related protein 5 polymorphism is closely associated with SLE susceptibility [13].

Although the relationship between autophagy and SLE has been continuously confirmed, there is still a lack of studies that systematically analyze the relationship between autophagy-associated genes and SLE. Which autophagy-related genes play a key role in SLE, how autophagy affects the immune process in SLE and whether disease diagnosis and classification can be made by autophagy status still need to be further investigated. In order to seek autophagy-related biomarkers in cSLE and achieve early and timely diagnosis, we obtained 7 disease signature genes (DDIT3, GNB2L1, CTSD, HSPA8, ULK1, DNAJB1, CANX) by constructing PPI network and Lasso regression analysis, and constructed artificial neural network models of 7 autophagy-related genes for diagnosis of cSLE, and the results showed that these biomarkers were effective for diagnosis of cSLE. Meanwhile, we validated the accuracy of this model in other datasets. To further investigate the immune cell infiltration in cSLE samples, we applied the CIBERSORT algorithm to calculate the immune cell content of cSLE samples, compared the immune cell differences between normal samples and cSLE samples, and analyzed the correlation between 22 immune cells and model construction genes. With this study, we hope to provide new perspectives for the investigation of autophagy-related genes in the diagnosis and mechanism of cSLE.

## Materials and methods

### Data download

We searched the GEO database (http://www.ncbi.nlm.nih.gov/geo/) for the keywords: "Children", "SLE". The relevant datasets were obtained and retained for those that met the following criteria: 1. whole gene transcriptome data; 2. human blood samples; 3. datasets with > 50 cSLE samples; 4. sample data completeness > 90%; 5. healthy reference groups were available. Finally, we retained the datasets GSE100163 and GSE65391. and obtained the complete transcriptome data of the datasets from GEO. The GSE100163 dataset was used as the training group, including 55 cSLE samples and 14 normal samples [14–16]; the GSE65391 dataset was used as the validation group, including 924 cSLE samples and 72 normal samples [17]. Also 232 autophagy-related genes were obtained from the previous top journals (Supplemental table 1).

### Variance analysis and enrichment analysis

We extracted the expression of 232 autophagy-related genes from the dataset GSE100163, and obtained 37 differential genes between SLE and normal samples using the R package "pheatmap" "limma" and plotted the heat map (| log2FC|> 1, FDR < 0.05). To further investigate the correlation between the differential genes, we plotted the correlation heat map (R package "corrplot") for the 37 differential genes. Meanwhile, the Gene Ontology (GO) and the Kyoto Encyclopedia of Genes and Genomes (KEGG) were performed on 37 differential genes using the R package "clusterProfiler", and the related bubble and histograms were plotted with *p*-value < 0.05.

### Screening of signature genes

We used the R package "glmnet" to perform Least absolute shrinkage and selection operator (LASSO) regression analysis on the differential genes to obtain genes that can distinguish SLE from normal samples. In addition, we constructed a Protein–protein interaction (PPI) network for the differential genes and selected the core genes associated with the disease. The disease-associated genes obtained by the above two methods were intersected to obtain the most valuable disease-characterized genes. Meanwhile, we compared the expression differences of these signature genes between SLE and normal groups, plotted the heat map of the differences, and showed the signature gene co-expression relationships using circle plots.

### Construction and validation of artificial neural network

The data set GSE100163 was used as the training group for the construction of the artificial neural network, and the R package "neuralnet" was used to construct the artificial neural network of disease characteristic genes, and four hidden layers were set as model parameters. The sum of the product of gene weights and expression levels of important genes was used as the basis for disease classification. Receiver Operating Characteristic curves (ROC, calculated AUC) plotted by the R package "pROC" "ggplot2" were used to assess the diagnostic efficacy of the artificial neural network in predicting the disease. In addition, the dataset GSE6539 was used to verify the accuracy of the constructed artificial neural network for the diagnosis of cSLE.

### Immunological correlation analysis

The immune cell content of cSLE and normal samples in the data set GSE100163 was obtained using "CIBERSORT", and the associated violin plot and histogram of immune cell content in each sample were plotted. Meanwhile, we plotted Lollipop charts to show the correlation between important genes constructed by artificial neural networks and immune cells.

### Statistical analysis

In the present study, statistical analyses were performed using R software (version 4.0.1). Depending on the type of data, the t-test and Wilcoxon rank-sum test were used in the analysis of quantitative variables. spearman's correlation was used to investigate the relationship between the expression of characteristic genes and immune infiltrating cells. *p* < 0.05 was considered to be statistically significant.

## Results

### Differential expression and enrichment analysis

**Fig. 1:**
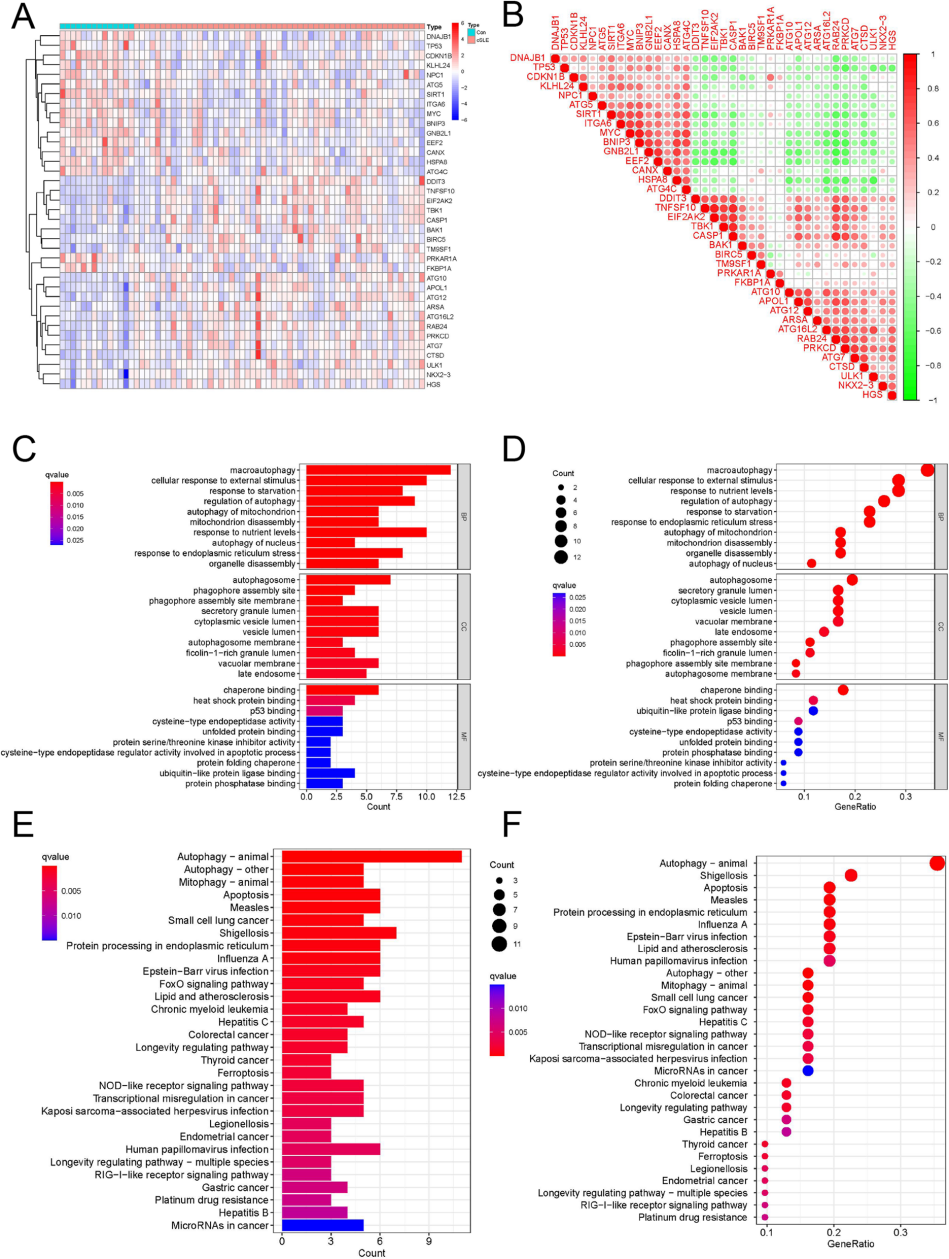
Differential analysis and enrichment analysis. **A** heat map of autophagy-related differential genes in normal and cSLE groups; **B** differential gene correlation plot; **C**-**D** histogram and bubble plot of differential gene GO enrichment; **E**–**F** histogram and bubble plot of differential gene KEGG enrichment

We analyzed the expression of 232 autophagy-related genes in the dataset GSE100163 and obtained 37 genes differentially expressed in normal samples and SLE according to the screening criteria |log2FC|> 1 and FDR < 0.05 (Fig. 1A), and also analyzed these gene correlations (Fig. 1B). To further understand the pathways and functions enriched by these differential genes, we conducted the Gene Ontology (Fig. 1C, D) and Kyoto Encyclopedia of Genes and Genomes (Fig. 1E, F). The results showed that in addition to autophagy related functions and pathways, these genes were also enriched in response to stavation, cellular response to external stimuli, mitochondrion disassembly, response to nutrient levels, apoptosis, shigellosis and protein processing in endoplasmic reticulum.

### Artificial neural network construction

**Fig. 2:**
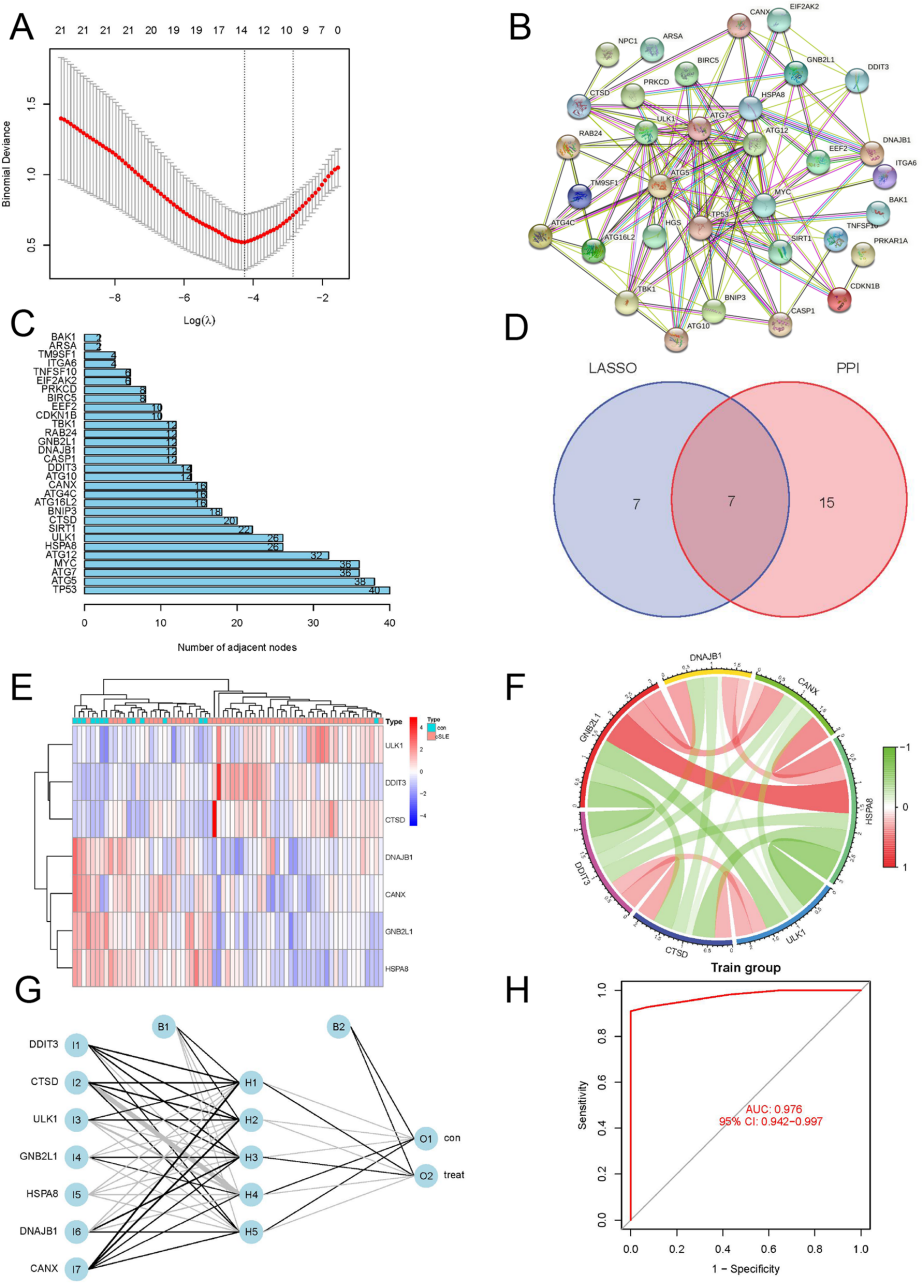
Construction of artificial neural network. **A** results of LASSO; **B** PPI network diagram of autophagy-related differential genes; **C** histogram visualizing the number of connected nodes within the PPI network of autophagy-related differential genes; **D** Venn diagram of LASSO and PPI screening of characteristic genes; **E** heat map of the difference in expression of characteristic genes in the normal and cSLE groups; **F** characteristic gene co-expression relationship of circle diagram; **G** Visualization of artificial neural network; **H** ROC curve of artificial neural network model for diagnosis of cSLE

To further find the characteristic genes associated with the disease, we performed Lasso regression on 37 differential genes for feature screening (Fig. 2A) and selected the 14 genes that best distinguished the disease (Supplemental table 2). Meanwhile, we constructed protein interaction networks for differential genes and showed the number of nodes for each gene (Fig. 2B, C). To avoid missing important genes, we treated genes with ≥ 10 node connections as important genes and obtained a total of 22 important genes (Supplemental table 2). The intersection of the important genes obtained by PPI and Lasso was taken (Fig. 2D), and seven overlapping genes were obtained (DDIT3, GNB2L1, CTSD, HSPA8, ULK1, DNAJB1, CANX). We used these seven genes as disease signature genes, and by differential heat map and co-expression circle map, we could find that CTSD, DDIT3, ULK1 were up-regulated in cSLE, GNB2L1, HSPA8, DNAJB1, CANX were down-regulated in cSLE (Fig. 2E), and there was a significant positive correlation between the expression of GNB2L1, DNAJB1, CANX and HSPA8 (Fig. 2F).

### Validation of artificial neural network

We constructed an artificial neural network diagnostic model for seven autophagy-related genes using the dataset GSE100163 (Fig. 2G). Firstly, we performed normalization preprocessing on the dataset. We set up 7 input layers with disease-characterizing genes, and in addition, 4 hidden layers were obtained. Using the sum of gene weights and the product of significant gene expression levels as the basis for classification, the resulting artificial neural network model classification scores were calculated as follows:$\mathrm{neuraHF}=\sum \left(\mathrm{Gene Expression}\times \mathrm{Neural Network Weight}\right)$. Next, we plotted the ROC curve of the model diagnosis to assess the predictive power and obtained an AUC value of 0.976 (Fig. 2H).

**Fig. 3:**
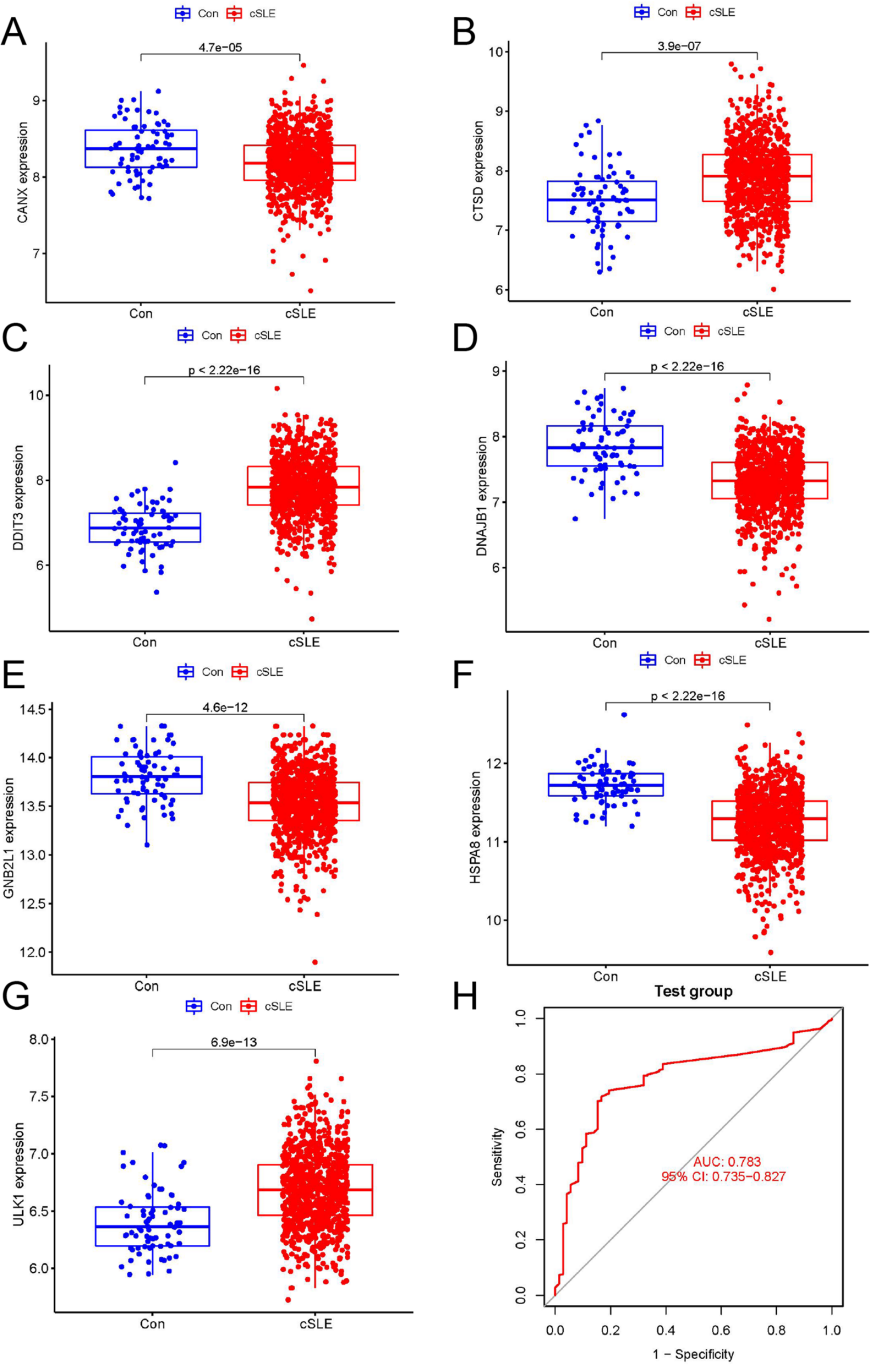
Validation of the artificial neural network model. **A**-**G** box plot of the difference in expression of feature genes between the cSLE group and the normal group in dataset GSE100163; **H** ROC curve of the artificial neural network model diagnosis in dataset GSE100163

To further validate the accuracy of our constructed model, we observed the expression differences of seven model genes in another dataset GSE65391. CTSD, DDIT3, and ULK1 expression was increased in cSLE, while GNB2L1, HSPA8, DNAJB1, and CANX expression was just the opposite, which was consistent with the results we obtained in dataset GSE100163 (Fig. 3A-G). Meanwhile, we plotted ROC curves to validate the model, and the results showed an AUC value of 0.783 (Fig. 3H), which indicated that the model we constructed exhibited good predictive power.

### Immune cell correlation analysis

**Fig. 4:**
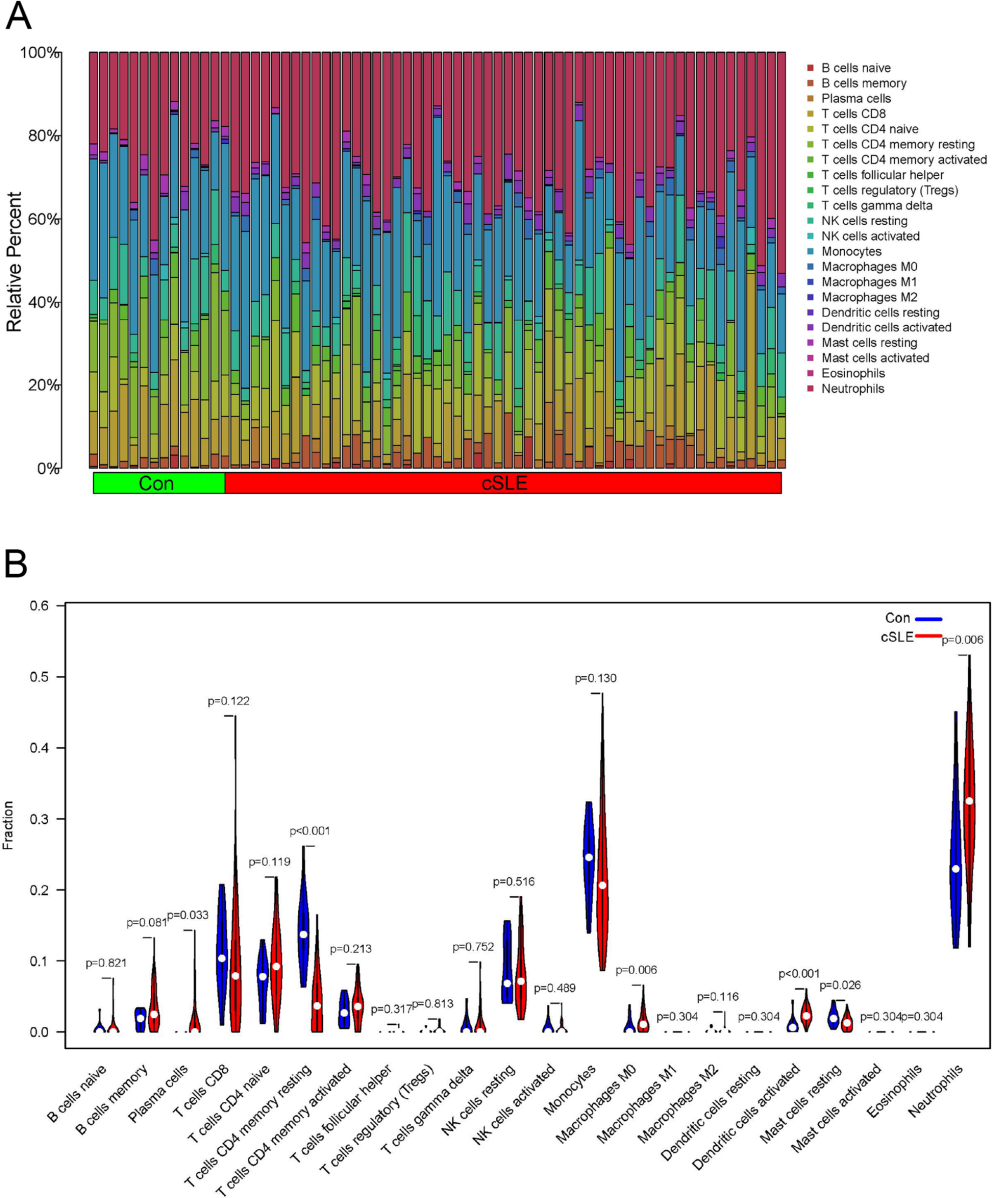
Immune infiltration analysis. **A** Histogram of the proportion of various immune cell infiltrations in the normal and cSLE groups; **B** violin plot of immune cell differences between the normal and cSLE groups

To understand the relationship between immune cell infiltration and cSLE, we calculated the immune cell content of each sample in the dataset GSE100163 using "CIBERSORT", and the results showed that Plasma cells, Macrophages M0, Dendritic cells activated and Neutrophils were more infiltrated in cSLE samples than in normal samples (Fig. 4A, B).

**Fig. 5:**
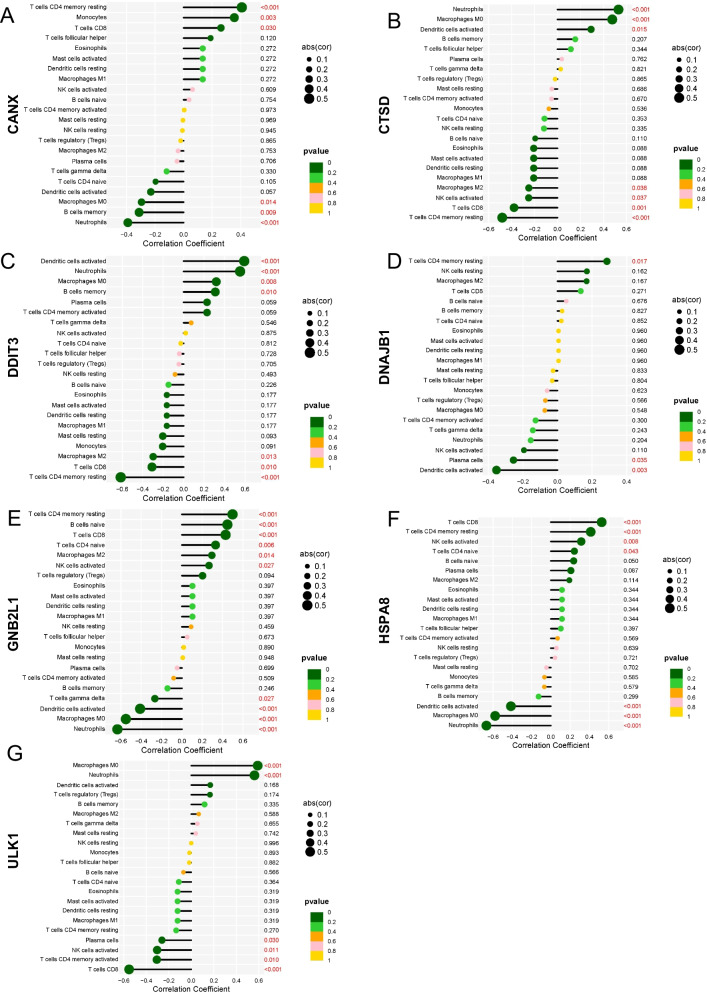
Relationship between signature genes and immune cells. **A**-**G** Lollipop charts of the correlation between signature genes and immune cells

Next, we analyzed the correlation of seven signature genes with immune cells. CANX was positively correlated with T cells CD4 memory resting, monocytes and T cells CD8 (Fig. 5A). CTSD was positively correlated with neutrophils, macrophages M0 and dendritic cells activated (Fig. 5B). DDIT3 was positively correlated with dendritic cells activated, neutrophils, macrophages M0 and B cells memory (Fig. 5C). DNAJB1 is positively correlated with T cells CD4 memory resetting.GNB2L1 was positively correlated with T cells CD4 memory resetting, B cells naive, T cells CD8, T cells CD4 naive, macrophages m2 and NK cells activated (Fig. 5D). HSPA8 was positively correlated with T cells CD8, T cells CD4 memory resetting, NK cells activated and T cells CD4 naive (Fig. 5E). ULK1 was positively correlated with macrophages M0 and neutrophils (Fig. 5F). These analyses demonstrate that key autophagy-related genes are closely associated with immune cell infiltration and play an important role in the immune mechanisms of disease development.

## Discussion

As a multisystemic disease with significant clinical heterogeneity, SLE has a complex pathogenesis and diverse clinical manifestations [18], which makes the diagnosis of SLE a great challenge. 70% of SLE patients have a long-term disease status between relapse and remission, which largely affects the quality of life of patients [19]. Autophagy, as an important defense mechanism, plays an important role in the pathogenesis of immune, infectious, inflammatory, tumor, cardiovascular, and neurodegenerative diseases of the organism [20]. In recent years, as the research on autophagy and SLE has intensified, the related mechanisms of action have been gradually elucidated. Autophagy is involved in multiple processes in multiple systemic diseases caused by SLE. It was found that autophagy mediates the release of TF-containing and IL-17A-containing NETs to participate in thrombosis and fibrosis in SLE [21]. Autophagy activators, on the other hand, prevent the destruction of podocytes in patients with lupus nephritis [22]. A study by Tania et al. found that autophagy-deficient SLE patients may result in a deficiency in the role of T lymphocytes [23]. In addition, it was also found that a defect in molecular chaperone-mediated autophagy (CMA) involving HSPA8 / HSC70, HSP90AA1 and lysosomal-associated membrane protein 2A (LAMP2A) was associated with SLE, and animal experiments showed that splenic LAMP2A expression was increased in purified B cells from lupus mice [24]. Concurrently, there have been relevant studies suggesting a treatment for SLE by affecting autophagy, and in a study for the treatment of cSLE, sirolimus was found to play a role in the treatment of SLE by modulating autophagy levels [25].

Our research compared the expression differences of autophagy-related genes between normal and cSLE samples, and seven autophagy-related genes (DDIT3, GNB2L1, CTSD, HSPA8, ULK1, DNAJB1, CANX) were screened to construct an artificial neural network model for diagnosis. The current study found that HSPA8 and ULK1 are associated with the pathogenic mechanism of SLE. HSP8A is one of the participants in the chaperone-mediated autophagy (CAM) process, and studies have shown that CAM is involved in SLE and that adjustment of over-activated CAM is beneficial for SLE [26]. ULK1 was significantly higher in patients with lupus nephritis type IV and V-IV than in normal controls (*p* < 0.05), and its involvement in autophagy-related pathways influences the pathological process of lupus nephritis [27]. whereas the relationship between the other 5 autophagy-related genes and SLE has not been elucidated. DDIT3 is a multi-stress condition-induced transcription factor localized in the nucleus and cytoplasm, involved in regulating cell cycle arrest and apoptosis and has been shown to promote chondrocyte autophagy via the SIRT1-AKT pathway [28, 29]. GNB2L1 functions in a variety of cellular activities and is involved in the recruitment, assembly and/or regulation of multiple signaling molecules [30]. CTSD, cathepsin D, is an acid protease that works on intracellular proteolysis and plays a dual role in cell proliferation and apoptosis [31]. DNAJB1 interacts with HSP70 to stimulate ATPase activity and also negatively regulates heat shock-induced HSF1 transcriptional activity [32]. CANX, a calcium-binding protein, interacts with newly synthesized glycoproteins in the endoplasmic reticulum and is required for endogenous pre-collagen autophagy-mediated quality control [33].

In conclusion, we screened seven autophagy-related genes closely associated with cSLE, among which CTSD, DDIT3 and ULK1 expression was significantly up-regulated in cSLE, while GNB2L1, HSPA8, DNAJB1 and CANX were significantly down-regulated. Meanwhile, the expression of these key genes influenced the immune infiltration of cSLE. In addition, the artificial neural network we constructed regarding these seven genes showed better value in diagnosing cSLE. Although the publicly available dataset of GEO is helpful in exploring key genes in cSLE, the role of these genes in cSLE is still unknown. The limitation of this experiment is the lack of sufficient samples similar to the GEO dataset to validate the expression of these key genes in cSLE, while animal experiments are still needed to explore the mechanism of action of key genes.

## Conclusion

We screened the seven most relevant autophagy-related genes in childhood SLE and constructed an artificial neural network model for diagnosis. We found that this model has good diagnostic efficacy and has been validated. We also found that these 7 key genes are closely related to the immune microenvironment of childhood SLE and may regulate the function of these immune cells to influence the development of the disease. We hope that this model can guide the development of clinical diagnosis and treatment, as well as provide a new perspective for exploring the pathogenesis of cSLE.

## Supplementary Information

**Additional file 1:** **Supplemental table 1.** 232 autophagy related genes.

**Additional file 2:** **Supplemental table 2.** LASSO and PPI acquisition of disease important genes.

## Data Availability

The data in this article are obtained from publicly available databases and are available from the authors.

## Abbreviations

CMA: Chaperone-mediated autophagy
cSLE: Childhood systemic lupus erythematosus
GEO: Gene Expression Omnibus
GO: Gene Ontology
KEGG: Kyoto Encyclopedia of Genes and Genomes
SLE: Systemic lupus erythematosus
LASSO: Least absolute shrinkage and selection operator
CMA: Chaperone-Mediated Autophagy
PPI: Protein–protein interaction
ROC: Receiver Operating Characteristic
